# Update on Innate Immunity in Acute Kidney Injury—Lessons Taken from COVID-19

**DOI:** 10.3390/ijms232012514

**Published:** 2022-10-19

**Authors:** Kinga Musiał

**Affiliations:** Department of Pediatric Nephrology, Wrocław Medical University, Borowska 213, 50-556 Wrocław, Poland; kinga.musial@umw.edu.pl

**Keywords:** coagulation cascade, complement system, immunothrombosis, necroinflammation, neutrophil extracellular traps, tubular epithelial cells, vascular endothelial damage

## Abstract

The serious clinical course of SARS-CoV-2 infection is usually accompanied by acute kidney injury (AKI), worsening prognosis and increasing mortality. AKI in COVID-19 is above all a consequence of systemic dysregulations leading to inflammation, thrombosis, vascular endothelial damage and necrosis. All these processes rely on the interactions between innate immunity elements, including circulating blood cells, resident renal cells, their cytokine products, complement systems, coagulation cascades and contact systems. Numerous simultaneous pathways of innate immunity should secure an effective host defense. Since they all form a network of cross-linked auto-amplification loops, uncontrolled activation is possible. When the actions of selected pathways amplify, cascade activation evades control and the propagation of inflammation and necrosis worsens, accompanied by complement overactivity and immunothrombosis. The systemic activation of innate immunity reaches the kidney, where the damage affecting single tubular cells spreads through tissue collateral damage and triggers AKI. This review is an attempt to synthetize the connections between innate immunity components engaged in COVID-19-related AKI and to summarize the knowledge on the pathophysiological background of processes responsible for renal damage.

## 1. Introduction

The clinical course of SARS-CoV-2 initially directed attention to influenza-like symptoms progressing into pneumonia and acute respiratory distress syndrome. The entrance of the virus through airways and its direct toxic impact suggested that its main influence was within the respiratory tract, with limited damage to other organs. Indeed, mild cases are usually restricted to an upper respiratory tract infection. However, meta-analyses have confirmed that the lower respiratory tract is not the only goal. Indeed, the kidneys are the second most commonly affected organ in patients hospitalized due to COVID-19 infection [1].

The renal involvement ranges in severity from proteinuria to acute kidney injury (AKI), defined according to KDIGO guidelines by serum creatinine increase and/or decrease in urine output [2]. However, if the new AKI definition by Ostermann et al. [3], combining functional and damage criteria, is taken into account, the overall incidence of AKI in COVID-19 patients is even higher (Table 1). Moreover, the data concerning children have elucidated the clinical challenge of AKI even in those who have had mild-to-moderate COVID-19 [4,5].

COVID-19 infection is triggered once the spike (S) SARS-CoV-2 protein is cleaved by a proteolytic enzyme TMPRSS2 (transmembrane serine protease 2), which uncovers the sequence able to bind to the angiotensin-converting enzyme (ACE)-2 receptor. The discovery of ACE-2 as a virus entrance gate highlighted the potential risk of damage to any organ containing these receptors, including proximal tubular cells in the kidney. Experimental data have proven the ability of SARS-CoV-2 to invade kidney cells and induce their injury, leading to increased collagen expression, elevated levels of extracellular matrix, and finally to interstitial fibrosis [6]. SARS-CoV-2 RNA expression is present in proximal tubular cells and podocytes, where COVID-19 infection-related genes are upregulated [6]. The increased activity of tumor necrosis factor (TNF)α and nuclear factor κB (NFκB) has been noticed in proximal tubular cells, as well as increased activity of (TNF)α, NFκB, and transforming growth factor (TGF)β in mesenchymal cells [6].

Additionally, the theory on lung–kidney crosstalk has highlighted hypoxia and inflammation as major anomalies responsible for AKI due to ischemia-reperfusion injury and damage of renal tubules. The latter may result from the in situ response to activity of both migrating and resident immune cells. Moreover, recent data have advocated for the unique and active role of tubular epithelial cells (TECs) as elements of innate immunity in AKI development [7].

In the meantime, the increasing incidence of thrombotic events in patients with COVID-19 infection has suggested the paramount systemic involvement of innate immunity, whereas overactivity of complement system elements and the presence of increased concentrations of inflammation markers in serum have made this puzzle even more complicated [8,9,10].

These facts have given credence to the role of SARS-CoV-2 as a trigger of systemic disease with kidney involvement, although the pathogenesis of the latter has not been fully elucidated.

## 2. Possible Scenarios of Kidney Involvement in SARS-CoV-2 Infection

Clinical data point at the prerenal causes as the most frequent mechanism of AKI in COVID-19 patients [11]. Thus, systemic hemodynamic instability is the most probable mechanism of AKI in the course of COVID-19 disease [12]. However, the multifactorial nature of kidney damage in the course of SARS-CoV-2 infection results from both virus-triggered conditions and clinical background [12,13].

**Figure 1:**
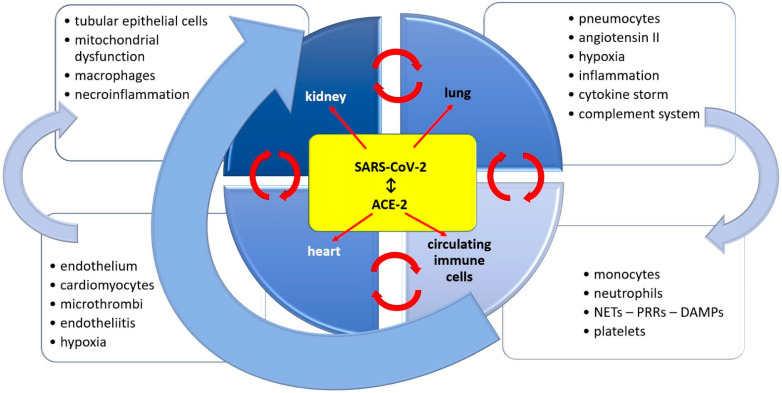
Interorgan and innate immunity cell crosstalk in COVID-19 infection. NETs—neutrophil extracellular traps; PRRs—pattern recognition receptors; DAMPs—damage-associated molecular patterns.

All AKI-related phenomena should be placed on a time scale, taking into account the progression from asymptomatic phase to multi-organ involvement pictured in [14]. The direct virus toxicity is responsible for the in situ endothelial damage, inflammation, complement system activation and coagulopathy [14]. The infection becomes symptomatic when indirect systemic effects add to the local conditions. The status of volemia, generalized infection or therapeutic approaches ease the systemic involvement and transform limited impact into generalized influence [14]. The organ crosstalk is also of paramount importance, adding to the innate immunity system and its circulating cells (Figure 1).

The molecular perspective points at hypoxia as a major contributor to the injury of kidney cells that may subsequently undergo uncontrolled necrosis. However, accumulating evidence has suggested that both non-programmed and programmed cell death modes may occur [15]. Although proximal tubules are most prone to injury due to their high metabolic activity, other structures within the kidney may also become involved. Direct virus toxicity may also affect podocytes, causing mitochondrial dysfunction. Endotheliitis and formation of microthrombi may add to vascular injury. Glomerular involvement, classified as COVID-19-associated nephropathy (COVAN), usually takes the form of collapsing glomerulopathy. Infiltration of immune cells within the kidney may trigger interstitial nephritis [15].

All the above-mentioned processes involve two major overlapping mechanisms of innate immunity: proteolytic cascades activating subsequent components of complement, coagulation and contact systems, as well as cellular responses generated by circulating immune cells and resident cells in the kidney. Interactions between these systems are mediated by inflammation and cell death.

## 3. Potential of Kidney Resident Immune Cells

Resident renal cells, originating from myeloid lineage, share the features of macrophages and dendritic cells, and thus are termed mononuclear phagocytes (MNP) [16]. This functional overlap of MNP and a variety of expressed surface markers allows them to act as sentinel cells ready for host defense against multiple pathogens. Their activity strongly depends on the external stimuli from the environment and the predominant type of cell death. In physiological conditions they add to the removal of cellular debris by releasing matrix metalloproteinases, diminish the number of circulating epitopes and maintain immune tolerance [17]. In the meantime, they present antigens to T cells and contribute to inflammatory reactions by the production of cytokines and chemokines [16]. TNF α release is the major event, since it triggers apoptosis and eases leukocyte extravasation through overexpression of adhesion molecules [17]. Such local adhesion molecule expression and chemokine production recruit immune cells circulating within the kidney blood vessels. As long as the balance between the generation of apoptotic cells and their phagocytic clearance is maintained, injury may resolve and AKI turns into the phase of repair [18]. This pathway is regulated by the colony stimulating factor (CSF)-1 through its receptor CSF-1R.

In the presence of pro-inflammatory IL-34, also acting through the CSF-1R receptor, unrestrained inflammation, combined with necrotic death, occurs. Necrosis activates resident macrophages which release proinflammatory molecules and trigger subsequent recruitment of neutrophils and monocytes from the circulation [19]. Further transition of monocytes into macrophages aggravates inflammation which in turn propagates necrosis and the in situ auto-amplification loop enables progressive renal tissue injury.

Mononuclear phagocytes also express elements of NLRP3 (NOD-like receptor family pyrin domain containing 3) inflammasome, acting as pattern recognition receptors (PRR) recognizing pathogen-associated molecular patterns (PAMPs), and damage-associated molecular patterns (DAMPs) released by cells undergoing programmed cell death. Detailed analysis of murine models with the use of modern techniques of 3D visualization has proven the activity of MNP cells in the conditions of LPS-induced AKI, as well as the renal function improvement with the use of MCC950—a specific NLRP3 inhibitor maintaining the activity of CD11c positive cells in an experimental model [20].

Tubular epithelial cells (TECs) per se take an active part in innate immunity defense too. The expression of scavenger receptors on proximal tubular cells allows them to gain the features of phagocytes. Moreover, TECs release DAMPs, that after recognition may activate NLRP3 and trigger secretion of proinflammatory cytokines. The activated inflammasome is able to release mature proinflammatory cytokines, triggering in turn further necrosis.

The major influence of TECs on innate immunity is exerted upon their necrosis with subsequent release of DAMPs. The presence of ACE2 receptors points to TECs as target cells in the kidney for SARS-CoV-2 invasion. Whereas the essential role of the spike (S) protein in the virus entrance into the cell is well described, a recent investigation has unraveled that another structural SARS-CoV-2 protein, nucleocapsid (N), is also responsible for evoking proximal tubule G1 cell cycle arrest through interaction with Smad3 and TGFβ/Smad3 signaling [21]. Irrespective of the pathogen type, the secretion of danger signals gives way to macrophage polarization towards a proinflammatory phenotype. One of the possible mechanisms was discovered recently, i.e., it leads, via the TLR2/caspase-5/Panx1 axis, to NLPR3 inflammasome activation in macrophages [22].

TECs’ immunometabolism, modified by insufficient energy supplies, probably redirects the response to stress either into reparatory phase or maladaptation leading to AKI-to-CKD transition, irreversible fibrosis and tissue damage. Moreover, ischemic damage to mitochondria within the tubules triggers the release of mitochondrial DNA, additionally activating innate immunity [23].

## 4. Circulating Immune Cells

Neutrophils and monocytes migrate to sites of infection and injury, attracted by the in situ macrophage production of cytokines [24]. The presence of surface receptors for adhesion molecules and cytokines enables the recruitment of these cells to the kidney, where injury triggers upregulation of ICAM-1, E- and P-selectin. The latter aggravates in situ inflammation and endothelial damage. Moreover, monocytes recruited into the injured kidney are activated in the course of binding PAMPs and DAMPs to PRRs and undergo differentiation into the proinflammatory phenotype of M1 macrophages. The subsequent production of proinflammatory cytokines and inducible nitric oxide synthase (iNOS) aggravates cytotoxic effects and, again, potentiates the in situ inflammation.

Neutrophils are the most abundant leukocytes in the circulation and form the first line of defense in innate immunity. Their phagocytic potential is based on the secretion of reactive oxygen species (ROS) and proteolytic enzymes, as well as on their ability to form neutrophil extracellular traps (NETs) [24]. Their primary role is to surround a pathogen, neutralize its virulence and kill it in a process called NETosis. However, recent data connect NETs to the formation of thrombi [25], activation of coagulation cascade [26] and multiorgan damage in COVID-19-induced sepsis [27].

COVID-19 patients present with heterogenous neutrophil populations, dominated by low-density granulocytes that show pro-inflammatory features, high production of cytokines and reactive oxygen species (ROS), increased phagocytic potential and the ability to produce NETs spontaneously [28,29].

Extensive NETs formation expels potential autoantigens, like double-stranded DNA or histones, into the extracellular space, which in turn may trigger autoimmune reactions. Additionally, neutrophil death releases DNA and proteases. All of these act as DAMPs and disseminate innate immunity activation [30].

Recent investigation has also provided evidence for platelet engagement in innate immunity [31]. Platelets expose TLRs and proinflammatory receptors CXCR4 and CXCR7 on their surface. Platelet alpha-granules contain bactericidal proteins, chemokines and P-selectin. Moreover, platelets take part in neutrophil stimulation to excrete neutrophil extracellular traps (NETs) [31]. In detail, infection triggers the release of platelet factors (e.g., platelet factor 4) and extracellular vesicles, activating neutrophils to secrete NETs [32].

This complex web of interactions between circulating immune cells amplifies the inflammatory reactions that, together with cell death, lead to collateral damage and subsequent systemic spread.

## 5. Programmed Cell Death in AKI

Uncontrolled cell death was first blamed for tubular injury in AKI, but recent experimental data have shown that also various types of programmed cell death, regulated by specific signaling pathways, contribute to the early phase of injury in acute tubular necrosis.

### 5.1. Necroptosis

Necroptosis is regulated by several signaling pathway proteins: receptor-interacting protein kinases (RIPK) 1 and 3, and their downstream target (mixed-lineage kinase domain-like—MLKL). Their role in AKI development has been tested in animal studies which showed that mice deficient in RIPK3 and MLKL were protected against ischemia-reperfusion injury [33,34]. Loss of plasma membrane integrity is the prerequisite to leakage of cell contents, swelling of organelles and release of DAMPs. The latter activate receptor NLRP3, which binds to its adapter molecule and becomes a complex activating caspase-1, termed inflammasome. The sequence of above-mentioned reactions within renal tubules initiates crosslinks between regulated necrosis and inflammation, and promotes irreversible damage and cell loss. Recent data have proven that RIPK3-MLKL-regulated necroptosis initiates early renal injury with NLRP3 inflammasome activation and subsequent acceleration of the necrosis-inflammation auto-amplification loop [35]. Therefore, this pathway may largely contribute to AKI-to-CKD progression.

### 5.2. NETosis

The formation of NETs may undergo either lytic/suicidal or non-lytic/vital pathway [30]. Lytic NETosis is induced by most known pathogens, including viruses, and results in cell membrane breakage and death. This disintegration drives calcium release, ROS production and activation of proteolytic enzymes that trigger decondensation of chromatin and cleavage of histones. The whole process is typical for the early phase of injury and lasts only for a few hours.

Non-lytic NET formation is driven by contact with activated platelets or in the course of infection triggered by Staphylococcus aureus [30]. It preserves the structure of the neutrophil membrane and maintains the integrity of the cell. Therefore, DNA may be transported in vesicles through the cytoplasm to the membrane and then outside the cell in order to form NETs. NETs formation components, such as elastase or myeloperoxidase, interact with B-lymphocytes, T-lymphocytes and antigen-presenting cells in order to secrete TNF α, IFNγ or interleukins (IL-6, IL-8). Thus, NETosis forms a natural link between necrosis and inflammation, aggravated by the reciprocal immunomodulatory effects of NETs on neutrophils. In vitro studies have shown that the exposure of human neutrophils to NETs has triggered ROS and NET production, as well as increased phagocytic and bactericidal potential of neutrophils [36].

The formation of NETs provides a first-line defense in the case of infection, so such a mechanism should be controlled effectively. However, clinical data suggest that excessive NETs production is a common finding during infections, including COVID-19 [37]. In detail, SARS-CoV-2 can trigger healthy neutrophils to release NETs and to stimulate NETosis aggravation [38,39]. NETs may enhance inflammation, collateral tissue damage and coagulation, leading to immunothrombosis in microvessels [40,41].

### 5.3. Ferroptosis

This recently depicted death pathway is iron-dependent and engages lipid peroxidation. Although not entirely unraveled, major mechanisms involve the failure of glutathione peroxidase (GPX)-4 activity. Experimental data have revealed acute renal failure in the case of its depletion in mice [42]. The original locus of ferroptosis discovery concerned renal tubules, most probably due to their high metabolic turnover [43]. Due to the high toxic potential of lipid peroxides, cells undergoing ferroptosis may not endure long enough to affect other components of the immune system. 

### 5.4. Pyroptosis

Contrarily to ferroptosis, pyroptosis bears the burden of high inflammatory impact on surrounding tissues and cells. The trigger factor is activation of caspase-11 by the inflammasome, together with the induction of proinflammatory cytokines. There is no proof for pyroptotic necrosis of renal cells, but it has been well documented in dendritic cells and macrophages that do appear in the kidney in the course of AKI.

## 6. Necroinflammation

Cells undergoing necrosis release proinflammatory alarmins, such as TNFα or IL-1α, and DAMPs [44]. The latter may originate from the nucleus (DNA, RNA), cytosol (e.g., heat shock proteins, SAP130) or mitochondria (mitochondrial DNA, ATP) [23,45]. As soon as they are released from dead cells, DAMPs alert the innate system through pattern recognition receptors (PRRs) located on the surface of other cells or intracellularly. The main four groups of PRRs are transmembrane Toll-like receptors (TLRs), C-type lectin receptors (CLRs), retinoic acid-inducible gene 1 (RIG1)-like receptors, as well as cytoplasmic nucleotide-binding and oligomerization domain (NOD)-like receptors (NLRs), with their flagship representative NLRP3 engaged in inflammasome activation.

The localization of PRRs on circulating immune cells, such as neutrophils, macrophages or dendritic cells, guarantees further release of proinflammatory mediators by these cells and propagation of necrosis through various pathways. TNFα induces the subsequent activation of RIPK3 and MLKL, whereas IFNγ acts through STAT3-protein kinase to activate necroptosis [46]. TNFα is also able to induce NETosis, whereas NLRP3 activation can trigger pyroptosis [24,47]. Thus, released cytokines enhance necrosis reciprocally.

This auto-amplification loop of necrosis and inflammation is termed necroinflammation [44]. The characteristic feature of this bidirectional causality is the limited intensity of the original process (like few necrotic cells), aggravated by the inflammatory spread (cytokine release), leading to collateral damage and amplification of the process by the subsequent death of the next cells, further inflammatory intensification, etc.

The existence of such an auto-amplification loop may at least partially dispel doubts about the disproportion between mediocre viral load in the kidney with COVID-19-related AKI, and the intensity of tissue damage with inflammatory/thrombotic sequelae. Probably, even a low level of viremia, triggering death of a limited number of tubular cells, may propagate through inflammatory reactions and turn collateral tissue damage into organ failure.

## 7. Complement System

Focus on the complement system in COVID-19 infection has resulted from clinical data connecting low C3 levels and high C5a levels with poor prognosis [48,49]. The mechanism of complement system activation is another example of coexisting proteolytic pathways leading to the same goal—formation of terminal membrane attack complex (MAC-C5b-9). Classical, lectin, and alternative pathways secure various options of host defense against pathogens. Spontaneous hydrolysis of C3 activates the alternative pathway, the classical pathway requires recognition by C1q, whereas the lectin pathway activation relies on the formation of a complex between mannan-binding lectin-associated serine protease (MASP)-2 and mannose-binding lectin (MBL), ficolin or collectin-11. However, the detailed description of pathways triggering systemic complement cascade activation during COVID-19 infection is beyond the scope of this manuscript and has been reviewed extensively elsewhere [50,51].

The proof for activation of a complement system in the renal tissue of patients with COVID-19 infection has been obtained from the human kidney biopsies performed in seven patients with SARS-CoV-2-related AKI [52]. While virus presence was sporadic, selected elements of the complement system were found in different localizations. C3 was the most frequent finding and its cleavage products (C3c, C3d) dominated in renal arteries. Peritubular capillaries hosted the activator of lectin pathway, mannan-binding lectin-associated serine protease-2 (MASP-2). The alternative pathway activity was focused on tubules, and the C1q typical for the classical pathway was negligible [52]. 

A more recent investigation, concerning 38 patients who died from COVID-19, has updated previous results and deciphered the dominant role of lectin pathway in the complement activation in the kidney. The deposition of complement factors was assessed in the lungs and kidneys of COVID-19 patients and of non-COVID-19 controls. MASP-2 was deposited in peritubular capillaries and tubular basement membranes, but was absent in renal arteries and glomeruli [53]. Kidney MASP-2 deposition was 10 times stronger in COVID-19 patients vs. controls, whereas that of C3 and C1q was weak. The alternative pathway activator factor D was markedly deposited in the lungs, contrarily to the kidneys [53].

Renal tubules were the most affected structures and the level of kidney injury correlated with the lung injury score. Surprisingly, SARS-CoV-2 spike protein was present only in 22% of lung tissue samples and in none of the kidneys. However, it was shown that recognition proteins of the lectin pathway are able to bind not only to SARS-CoV-2 S-, but also to N-proteins [54].

Thus, the virus presence is not obligatory to propagate renal tissue damage in situ—it was the complement activation that makes it systemic.

Connections between the complement system and NETs may speak in favor of such a scenario. In C3 knockout mice undergoing ischemia-reperfusion injury, kidney damage was attenuated, infiltration of neutrophils was lessened and NETs formation was diminished [55]. C5a was also able to stimulate in vitro NETs generation when added to human neutrophil cultures [56]. Moreover, it triggered the increased production of mitochondrial ROS. An animal study revealed that selective C5aR1 receptor could diminish the size of the thrombus and decrease NETs formation in mice with left common carotid artery thrombosis [56]. Additionally, in vitro experiments have proven that the exposure of neutrophils from healthy volunteers to complement C5a results in the shedding of proinflammatory microvesicles and loss of C5a receptor (C5aR)1 on neutrophils [57].

Interestingly, the spectrum of complement components on circulating innate immunity components from COVID-19 patients differed from the in situ renal cell composition. In detail, the upregulation of C1q and C3 on monocytes underlined the activity of the classical pathway, whereas overactivity of CD55, a complement inhibitor, highlighted the systemic compensatory mechanisms during COVID-19 infection [58].

## 8. Complement–Coagulation Interactions

The coexistence of complement overactivity and thrombotic events in patients with COVID-19 reminds us of the tight connections between these two systems [40,41]. The clinical significance of coagulation factors increased when it became clear that they may serve as biomarkers of COVID-19 infection severity and patient outcome [59,60].

These crosslinks are a natural consequence of the fact that vascular endothelial damage is a prerequisite for the activation of coagulation cascade. The availability of the subendothelial layer not only exposes active players in the clot formation, such as von Willebrand factor or collagen, but also triggers activation of pro-coagulative tissue factor (TF) and excessive production of thrombin. However, the impact of coagulation on COVID-19 infection severity begins at the entrance of the virus into the cell. TMPRSS2, the priming spike protein for fusion through ACE-2, can be inhibited by antithrombin, disabling virus invasion and cell damage [61].

Additionally, angiotensin-converting enzyme (ACE)-2 itself is of paramount importance in triggering mechanisms leading to kidney injury and systemic changes in the course of COVID-19 infection. ACE-2 activity links the renin-angiotensin and kallikrein-kinin systems. The cleavage of angiotensin II into Ang 1-7 by ACE-2 exerts anti-inflammatory, anti-oxidant and anti-fibrotic effects, similar to the result of cleavage of bradykinin 1-8 into bradykinin 1-7 [62]. One intriguing hypothesis suggests that internalization of SARS-CoV-2 makes ACE-2 receptor less available and the intensity of the above-mentioned protective proteolytic reactions is diminished. Thus, angiotensin II may act in a destructive way through its receptor AT1, activating terminal components of the complement system (C5a, C5b-9). Moreover, Ang II enables cytokine release, stimulation of nuclear factor kappa B (NF-κB), TNFα or IL-6, triggering inflammatory reactions, necrotic sequelae with NETs release and auto-amplification loops of necroinflammation.

Moreover, histones released during NETosis act toxically versus endothelial cells, trigger activation of platelets and further thrombosis [63,64]. Platelets aggravate thrombin generation and thrombus formation [65]. NETs promote death of tubular epithelial cells and also subsequent processes such as clotting in the peritubular capillaries. Activated platelets secrete cytokines and chemokines that further recruit immune cells, including leukocytes, to the sites of injury, and trigger endothelial cell activation. Microvascular injury aggravates thrombosis, inflammation accelerates necrosis and all these phenomena create another auto-amplification loop within the spectrum of AKI.

## 9. AKI in the Omicron Era

The systematic appearance of new SARS-CoV-2 variants of concern (VOC) raises questions about the differences in clinical course, organ involvement and prognosis. The first results of comparisons of the disease severity between Delta (B.1.617.2) and Omicron (B.1.1.529) variants pointed out the lower risk of hospitalization, better in-hospital outcomes and lower mortality in patients infected with Omicron [66,67,68]. Surprisingly, none of these studies has compared the incidence of AKI in both groups.

There is, in fact, only one publication analyzing the Delta–Omicron disparities concerning AKI in critically ill patients. Of note, the results have to be interpreted cautiously because of the small study group [69]. The comparison of 31 Omicron patients and 34 Delta patients revealed higher mortality rates and numbers of pre-existing comorbidities, as well as more frequent AKI incidence, in those who suffered from Omicron variant infection. Apparently, some of these discrepancies can be explained by the higher rate of patients with positive vaccination status among those with Omicron vs. Delta infection. Thus, although the overall incidence of admissions to intensive care units tended to decrease in Omicron patients, those who required hospitalization suffered from more preexisting comorbidities and presented with a more severe clinical picture.

The paradox of higher AKI prevalence in Omicron vs. Delta patients may result from faster virus replication for the price of less specialized cell tropism, priming the spreading of infection in the population over preference to specific organs [69]. Such low specialization with ubiquitous access would also add to the overall severe clinical status of Omicron patients in the ICU. However, this preliminary observation requires verification in a larger group of patients.

## 10. COVID-19 Infection in Patients with Preexisting Kidney Injury

Clinical reasoning suggests that preexisting renal damage, from AKI through acute kidney disease (AKD) to chronic kidney disease (CKD), may worsen the course of COVID-19 infection, although currently there is no proof for this hypothesis in the literature. Infection in immunocompromised patients always carries the risk of AKI-to-AKD and AKD-to-CKD transition. Limited data on small groups of patients show that preexisting CKD is responsible for a more serious clinical course, increased mortality and timely kidney function deterioration in COVID-19 patients [70]. However, a meta-analysis has pointed out the increased prevalence of preexisting CKD in severe vs. non-severe COVID-19 patients, but did not confirm CKD progression in the course of SARS-CoV-2 infection [71]. Therefore, underlying CKD aggravates the COVID-19 clinical course, but the reciprocal reaction is not evident. No doubt, further research is required to highlight the SARS-CoV-2–renal injury crosslinks in regard to kidney function deterioration after the infection. 

One fact is, however, undeniable—the diminished effectiveness of vaccinations against COVID-19 in the population of patients with CKD, in those on renal replacement therapy, and, above all, in those after kidney transplantation [72]. Organ transplant recipients present with improved seroconversion no sooner than with the third vaccine dose.

## 11. Current Progress in COVID-19 Infection Prevention and Treatment

The detailed description of recent developments in antiviral drugs and vaccine types used for COVID-19 treatment and prevention is beyond the scope of this manuscript, but multiple excellent reviews have dealt with these subjects [73,74,75]. This area of research is a matter of the utmost importance, because SARS-CoV-2 has not yet revealed the full spectrum of its multifaceted nature. Undoubtedly, new variants are just around the corner, ready to test patients’ and scientists’ flexibility, but also to give stimulus to new discoveries in the area of effective prevention and therapy [76].

Taking into account the focus of this review on the innate immunity-related processes, anti-inflammatory drugs and selected mononuclear antibodies deserve mentioning as examples of discrepancies between theoretical knowledge on COVID-19 infection background and its translation into clinical practice. The results of clinical trials testing the efficiency of classical (glucocorticoids) and new generation anti-inflammatory drugs (Janus kinase inhibitor baricitinib, IL-6 receptor inhibitors tocilizumab and sarilumab) were rather disappointing. Their ability to limit the disease progression was confined and they did not reduce the mortality rate [77]. Additionally, none of these therapies could improve the kidney function.

Contrarily, complement system inhibition gave spectacular results of clinical improvement and decrease in mortality [78,79]. However, the impact on kidney function, especially on AKI progression/recovery, has not been analyzed yet.

## 12. Perspectives

The COVID-19 pandemic has been the major stimulus for scientific activity worldwide and, among many other topics, has regained due interest to acute kidney injury. The major task for the future is to define the pathogenic mechanisms in the early phase of infection in order to prevent its spread, AKI itself, and multiple organ damage with fatal prognosis. In order to achieve this goal, detailed analysis of the cross-talks between different organs, as well as between various elements of innate immunity within the kidney, is required.

Molecular insights into AKI also give the opportunity to advocate for the introduction of an extended definition of AKI into clinical practice. Subclinical AKI, with increased markers of tubular damage and preserved glomerular filtration, allows us to recognize the patients at risk of kidney damage earlier [3,80]. Unfortunately, this early phase is not taken into account in any of the recommended AKI classifications. However, there is still hope that the microscale arguments turn into macroscale modifications due to better understanding of destructive mechanisms within the kidney.

## 13. Conclusions

Innate immunity forms the web of finely tuned chain reactions with a defined number of connecting points, where various pathways may interact. The balanced response of selected elements of this network secures adequate defense against pathogens. The conditions leading to acute kidney injury in the course of SARS-CoV-2 infection seem to simultaneously activate all elements of host defense with the subsequent functional overload, exaggerated auto-amplification and “overheating” of the whole system. The COVID-19 pandemic has sharply unraveled how fragile the balance between various elements of innate immunity can be, but has also taught us that the update to AKI 2.0 is attainable.

## Figures and Tables

**Table 1:** New definition of acute kidney injury (AKI) (according to [3], modified).

Stage of AKI	Functional Criteria		Damage Criteria
	Serum Creatinine	Urine Output	
**1S**	No change or increase <0.3 mg/dl	No criteria	Biomarker (−)
**1A**	Increase by ≥0.3 mg/dL for ≤48 h or ≥150% for ≤7 days	<0.5 mL/kg/h for >6 h	Biomarker (−)
**1B**			Biomarker (+)
**2A**	Increase by ≥200%	<0.5 mL/kg/h for >12 h	Biomarker (−)
**2B**			Biomarker (+)
**3A**	Increase by ≥300% ( ≥4 mg/dL with an acute increase of ≥0.5 mg/dL) and/or acute renal replacement therapy	<0.3 mL/kg/h for >12 h or anuria for >24 h	Biomarker (−)
**3B**			Biomarker (+)

## Data Availability

Not applicable.
